# Tumor Angiogenesis after Heated Lipiodol Infusion via the Hepatic Artery in a Rabbit Model of VX2 Liver Cancer

**DOI:** 10.1371/journal.pone.0061583

**Published:** 2013-04-24

**Authors:** Wei Cao, Xiang Xu, Juliang Zhang, Yunyou Duan

**Affiliations:** 1 Department of Interventional Radiology, Tangdu Hospital, The Fourth Military Medical University, Xi’an, China; 2 Department of Pharmacy, School of Medicine, The Second Affiliated Hospital, Zhejiang University, Hangzhou, China; 3 Department of Vascular and Endocrine Surgery, Xijing Hospital, The Fourth Military Medical University, Xi’an, China; 4 Department of Ultrasonography, Tangdu Hospital, The Fourth Military Medical University, Xi’an, China; Northwestern University Feinberg School of Medicine, United States of America

## Abstract

**Objectives:**

This study aimed to observe the changes in tumor angiogenesis after heated lipiodol (60°C) infusion via the hepatic artery in a rabbit model of VX2 liver cancer.

**Materials and Methods:**

Twenty rabbits with VX2 hepatic tumors were randomly divided into 2 groups (10 rabbits in each group). Under anesthesia, a trans-catheter hepatic arterial infusion was performed, and lipiodol (37°C; control group) or heated lipiodol (60°C; treated group) was injected into the hepatic arteries of the animals. Then, changes in tumor angiogenesis were assessed using the following markers and methods. 1. Vascular endothelial growth factor receptor (VEGFR) and vascular endothelial growth factor (VEGF) expression levels in the tumor were assessed using western blotting and real-time quantitative polymerase chain reaction (PCR). 2. Proliferating cell nuclear antigen (PCNA) expression in the tumor was assessed through immunohistochemical staining. 3. The morphological changes in tumor vascular endothelial cells were observed using transmission electron microscopy (TEM).

**Results:**

VEGFR and VEGF mRNA and protein expression levels were reduced in the treated group compared to the control group. PCNA protein showed reduced expression levels in the treated group compared to the control group. TEM indicated that the endothelial cell endoplasmic reticulum expanded, the chondriosome was swollen, and the endothelial cell microvilli were decreased after heated lipiodol infusion.

**Conclusions:**

The tumor angiogenesis of rabbits with VX2 cancer was inhibited after arterial heated lipiodol infusion compared to lipiodol infusion.

## Introduction

Transcatheter chemoembolization (TACE) has been accepted as one of the most effective forms of palliative treatment for patients in the middle and late stages of hepatocellular carcinoma (HCC) as well as for those who are not good candidates for surgery in Asian countries, including China [1], [2], [3]. However, long-term survival after a TACE procedure is not satisfactory; the five-year survival rate currently ranges from 9% to 32% [4], [5]. TACE can reduce tumor size [6], [7]. However, other studies have found TACE to be unsatisfactory because tumor angiogenesis may increase after TACE, and only a small proportion of HCCs underwent complete necrosis [8]. Therefore, inhibiting tumor angiogenesis after TACE therapy may be a promising strategy to improve treatment efficacy.

Recently, some studies have used thermotherapy to suppress tumor growth in the liver [9] and have verified that treatment with lipiodol at 60°C prolongs the survival of rabbits with VX2 cancer by inhibiting tumor growth. In addition, this treatment affects serum AST levels similar to lipiodol at 37°C [10]. In vivo studies have also demonstrated that heated saline infusion via the hepatic artery can alter the tumor vascular permeability by affecting the expression levels of VEGF or VEGFR [11], [12] because these two proteins are two important factors related to tumor angiogenesis [13], [14]. Therefore, the aim of this study was to observe the changes in tumor angiogenesis and analyze the underlying causes after trans-hepatic, arterial, heated (60°C) lipiodol embolization via the hepatic artery in a rabbit model of VX2 liver cancer.

## Materials and Methods

### Ethics Statement

All animal experiments were performed in accordance with the protocol approved by our institutional animal care and use committee (2010038) and in compliance with institutional guidelines.

### Animal Models

Twenty male New Zealand white rabbits (weighing 3.0–3.5 kg, average 3.2±0.2 kg) were selected randomly from the experimental animal center of our university. VX2 carcinoma cells were maintained as a tumor line in our laboratory.

The rabbits were anesthetized with intravenous sodium pentobarbital (25 mg/kg). Next, the hairs over the abdominal region of the rabbits were removed with 8% sodium sulfide, and the region was cleaned with saline. A suspension of VX2 tumor tissue (approximately 1.5–2×10^6^ cells) was injected via an 18-gauge needle into the left lobe of the liver percutaneously with ultrasound guidance. The wound was kept sterile, and the rabbit vital signs (especially the rate of respiration) were closely monitored during the implantation. Next, antibiotics (gentamicin 2.5 mg/kg) were injected intramuscularly after the implantation. The animals were observed by sonography (Acuson Corp, USA) until the tumors reached 2 cm in diameter and were then used for experimentation. Ultrasonography was performed by the same operator with a 7v3c probe at a frequency of 7.0 MHz.

### Experimental Grouping

Twenty tumor-bearing rabbits were randomly divided into 2 groups (n = 10 per group); each group was given one of two preparations.

The control group received a preparation of lipiodol (Aulnay Sous-Bios, France) at physiologic temperature (37°C).

The treatment group received a preparation obtained by heating lipiodol to 60°C with a constant temperature incubator.

### Arterial Catheterization

An incision was made into the anesthetized rabbits to expose their right femoral artery. Then, a micro-catheter (2.7/2.9 Fr Reshapable Type, Terumo, Japan) was inserted into the femoral artery and positioned into the hepatic artery under fluoroscopy.

Next, under fluoroscopy, the perfusion preparation (1 mL/body) for each group was gradually hand-injected into the hepatic artery, with great care to keep the catheter tip in the hepatic artery and avoid efflux of the preparation. After intra-arterial injection, the catheter was removed, the femoral artery was ligated, and the operative wound was closed. The rabbit’s vital signs, especially its respiration rate, were closely monitored during the angio-procedure. DSA images of the VX2 liver tumor staining were acquired before and after the infusion.

### Animal Sacrifice

At 24 hours after preparation infusion, the animals were sacrificed with an overdose of chloral hydrate.

### Immunohistochemistry

Tumor tissue samples from each rabbit were fixed in 10% neutral buffered formalin and embedded in paraffin. Then, 3- to 5-µm-thick slices were cut and processed for immunohistochemical staining.

To detect the expression and location of PCNA protein in tumor tissues, the sample sections were incubated overnight at 4°C with a mouse anti-PCNA (Abcam, Cambridge, UK) monoclonal antibody and then with an anti-mouse secondary antibody (Dako) for 30 min at room temperature, after which the binding reactions were visualized with 3-30-diaminobenzidine tetrahydrochloride (DAB) substrate. The nuclei were lightly counterstained with hematoxylin. The immunohistochemical staining was evaluated by 2 pathologists in a blinded manner. The entire section of each slide was examined under light microscopy. The PCNA protein levels were graded into four groups: 0, no staining; 1+, cytoplasmic staining in less than 10% of cells; 2+, cytoplasmic staining in 10–50% of cells; and 3+, cytoplasmic staining in more than 50% of cells.

### Real Time Quantitative Polymerase Chain Reaction

The tumor samples were collected and stored in RNAwait (Applygen, Beijin, China) at −70°C after the rabbits were sacrificed. Total RNA was isolated from the samples with TRIzol reagent (Invitrogen, Carlsbad, CA), and cDNA was synthesized from 1 µg of total RNA with the First-Strand cDNA Synthesis Kit (Toyobo, Osaka, Japan). The oligonucleotide primers for polymerase chain reaction (PCR) were designed as follows: VEGF, 5′-GCAGAAGAAGGAGACAATAAACC-3′ and 5′-GCACGCAGGAAGGCTTGAATA-3′; VEGFR 5′-CCAGGGAGAAGCAGAGCCAC-3′ and 5′-TGCCAATACCAGCGGATGTG-3′; and β-actin, 5′-CGAGATCGTGCGGGACAT-3′ and 5′-CAGGAAGGAGGGCTGGAAC-3′. The PCR reactions were performed in triplicate using SYBR Green Real-time PCR master mix (Toyobo). The relative expression levels of VEGF mRNA were quantified using the 2^−ΔΔCt^ method [15].

### Western Blotting

The samples were lysed in ice-cold lysis buffer (2 mM EDTA, 10 mM EGTA, 0.4% NaF, 20 mM Tris-HCl, 1% NP-40, 1% Triton X-100, protease inhibitors, pH 7.5) using a glass homogenizer. The protein concentrations were measured using the bicinchoninic acid (BCA) protein assay (Pierce, Rockford, IL). The proteins were separated by sodium dodecyl sulfate polyacrylamide gel electrophoresis (SDS-PAGE) and transferred to Hybond ECL nitrocellulose membranes (Amersham Biosciences, Piscataway, NJ). Western blot analyses were performed using an anti-VEGFR antibody (Abcam, Cambridge, UK) and an anti-VEGF antibody (Santa Cruz, CA, USA), and an anti-β-actin antibody was used as internal control. Then, the samples were incubated with a species-matched horseradish peroxidase (HRP)-conjugated secondary antibody. The blots were developed with a chemiluminescence substrate solution (Pierce, Rockford, IL) and exposed to X-ray film. The IDVs were calculated using a computerized image-analysis system (Fluor Chen 2.0) and normalized to the expression of β-actin.

### Transmission Electron Microscopy (TEM)

After the tumor tissues were obtained, crude microvessel fractions isolated from the tumor tissue were selected and divided into 1 mm^3^ pieces and fixed with 2.5% glutaraldehyde–4% paraformaldehyde for several days at 4°C. Then, using standard procedures, semi-thin and ultra-thin sections were prepared and stained with uranyl acetate and lead citrate, and changes in the tumor vascular endothelial cells were observed using TEM (JEM-1200EX, Jeol, Tokyo, Japan).

### Statistical Analyses

All results are denoted as the mean ± SD for each group. The Student’s t-test was performed to compare the two groups, and the Mann-Whitney’s U-test was used to compare PCNA protein levels between groups. A P value of less than 0.05 was considered significant. Statistical analyses were performed using SPSS 16.0 for Windows (SPSS Inc., Chicago, IL, USA).

## Results

### DSA Image

**Figure 1:**
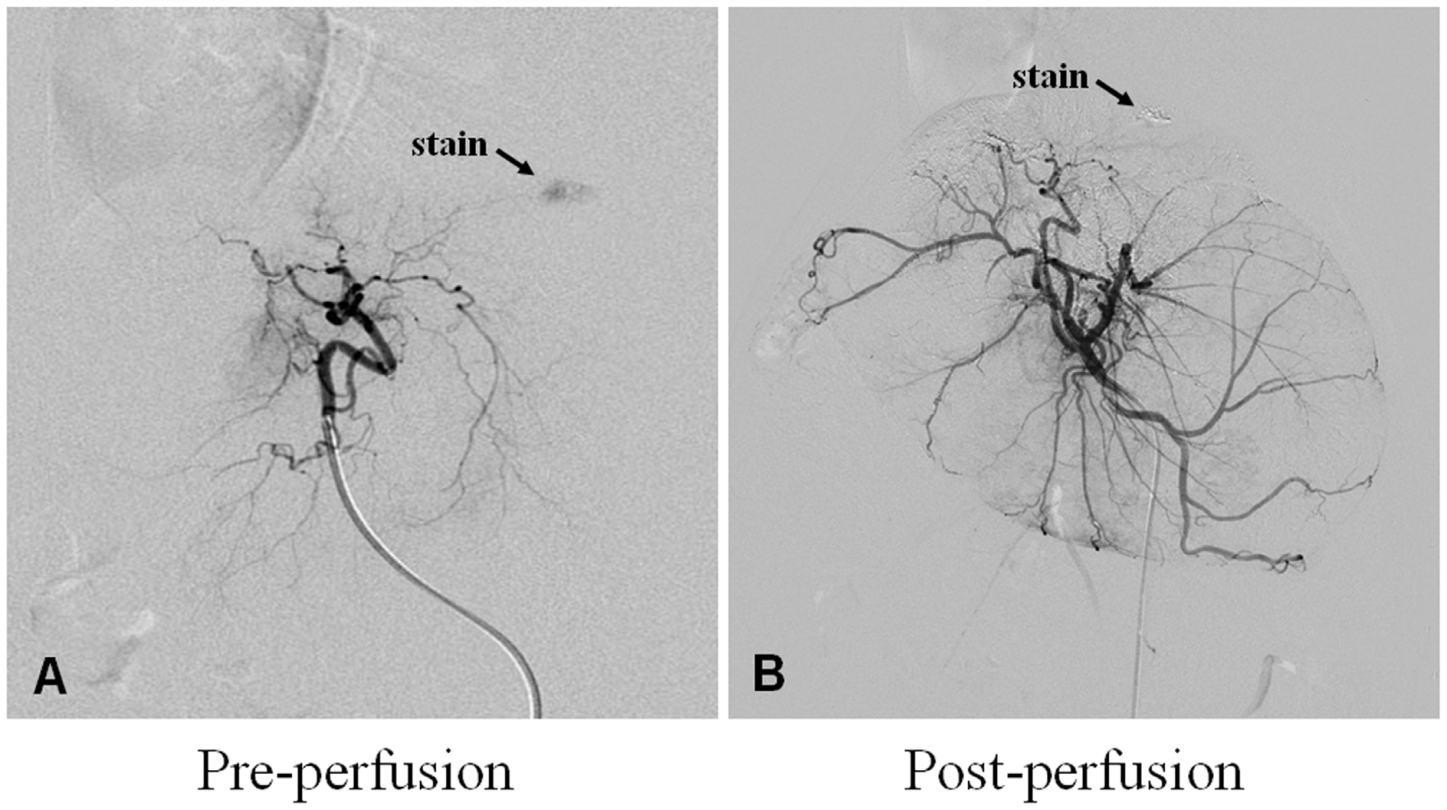
DSA imaging of treated tumors. The tumors showed hypervascularity in the liver as determined with DSA imaging (A, black arrow). After lipiodol (60°C) injection, the tumors are completely or largely de-vascularized and show on DSA as a lipiodol-filling defect (B, black arrow).

After embolization, the liver DSA images of the rabbits in the lipiodol (60°C) group showed contoured opacity in the tumor region, a sign of complete occlusion of the tumor vasculature, and the proximal normal vessels of rabbits still remain patent (Fig. 1).

### Immunohistochemical Findings

**Figure 2:**
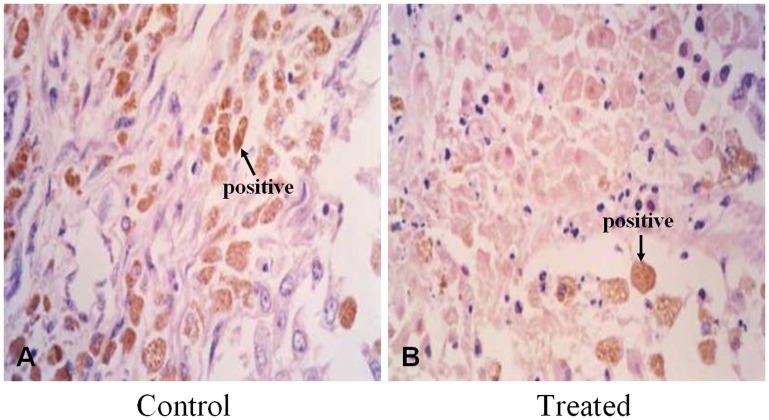
Expression of PCNA protein. As detected through immunohistochemistry, PCNA protein expression was detected mainly in viable VX2 tumor cells (brown; A, control 400**×**; B, treated 400**×**).

Immunohistochemical analyses revealed that PCNA protein expression was detected mainly in viable VX2 tumor cells (Fig. 2). We found that PCNA expression levels in the treated group were lower than in the control group (Table 1).

**Table 1:** Comparisons of PCNA protein levels between groups after perfusion.

Group	PCNA protein levels			
	0	1+	2+	3+
Control (37°C; *n* = 10)Treated (60°C; *n* = 10)	00	48	32	30

PCNA: control *vs.* treated group, *P* = 0.047.

### Real-time Quantitative PCR and Western Blot Findings

**Figure 3:**
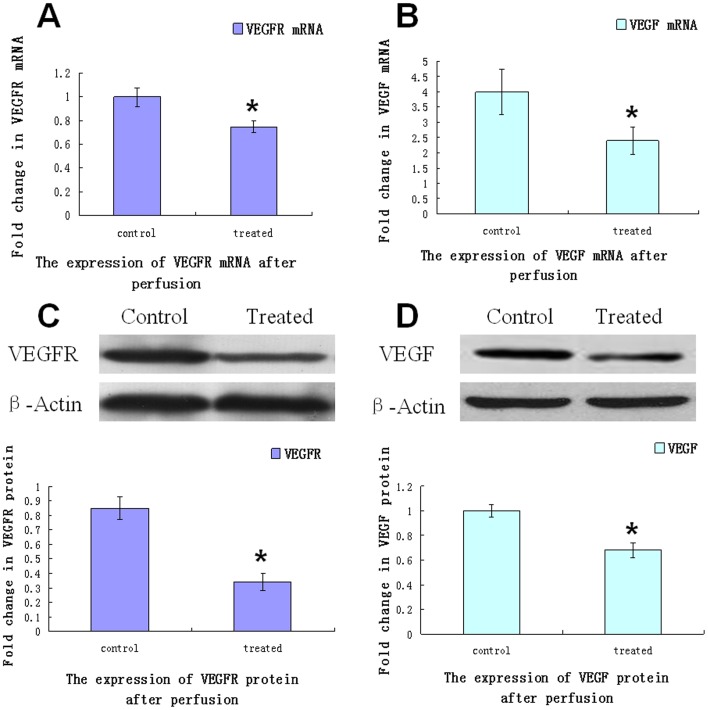
Expression of VEGFR and VEGF protein and mRNA levels. The relative changes in VEGFR or VEGF protein and mRNA levels in tumor tissue were detected after treatment in each group (n = 10). A and B. VEGFR and VEGF mRNA expression levels were evaluated using real-time quantitative PCR as described in the Materials and Methods section. C and D. VEGFR and VEGF protein levels were detected using Western blot analysis (upper panel). β-actin was detected as a loading control. VEGFR and VEGF expression levels were quantified through densitometry and plotted as the fold change (lower panel). The values are presented as the mean±SD of 3 independent experiments (* *P*<0.05 *vs.* control group).

As detected by real-time quantitative PCR (Fig. 3), VEGF and VEGFR mRNA levels in the treated group were significantly lower than in the control group. Western blot analyses showed that the expression levels of the VEGFR and VEGF protein in the treated group were also significantly lower than in the control group (Fig. 3).

### TEM Findings

**Figure 4:**
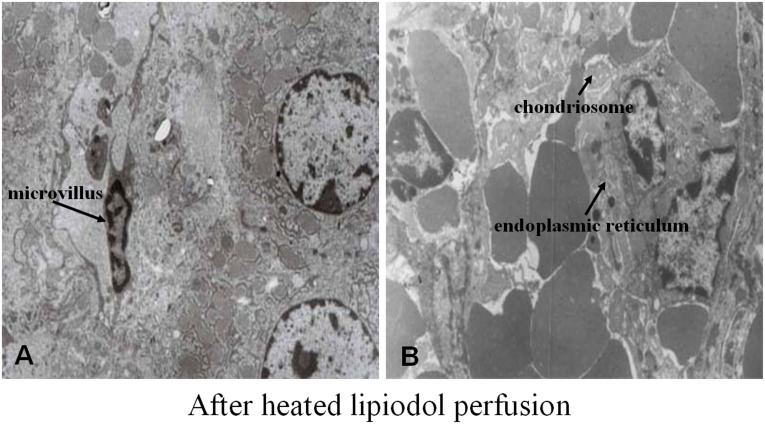
TEM results of treated tumors. Stimulated by heated (60°C) lipiodol perfusion, TEM results revealed that the tumor endothelial cell microvilli decreased (A, 5000×), the vascular endothelial cell endoplasmic reticulum expanded, and the chondriosome was swollen (B, 10000×).

TEM results revealed that the heated lipiodol perfusion induced the vascular endothelial cell endoplasmic reticulum to expand and the chondriosome to swell, and the endothelial cell microvilli were decreased (Fig. 4).

## Discussion

TACE for tumor de-vascularization was developed in the 1970s [16]. Currently, TACE is the most widely used primary treatment for unresectable HCCs and is also the most extensively used therapy for patients on the waiting list for liver transplantation. TACE significantly delays tumor progression [17], but a few studies have reported an increase in serum VEGF levels after TACE [18]. The high VEGF levels 1–2 days after TACE in HCC patients are associated with distant metastasis and unfavorable outcomes [19].

Lipiodol has been used as a carrier of anti-tumor agents in targeted chemotherapy for hepatocellular carcinoma, and lipiodol is not only used to emulsify drugs for TACE, but also used as an embolization agent for trans-hepatic arterial embolization and for tumor de-vascularization [17]. Because the VEGF protein expression in tumors may respond to heat [11] and the viable tumor vascular endothelial cells are sensitive to heat injury [20], and mass spectrometric analysis of drug showed that cisplatin is not affected by heat [21], and doxorubicin and mitomycin-C are also not degraded at temperatures of 60 degrees C [21], heated lipiodol as a new embolic agent via hepatic artery administration can distribute heat and emulsify conventional TACE drugs (doxorubicin, mitomycin-C, cisplatin) to the entire tumor and may affect VEGF protein expression levels in the tumor or the tumor vasculature.

### Preventing Tumor Angiogenesis After Heated Lipiodol Injection

After heated lipiodol injection, TEM revealed that the tumor endothelial cell endoplasmic reticulum expanded, the chondriosome was swollen, and the endothelial cell microvilli were decreased, which are signs of endothelial cell damage. These signs, although indirect, support the heat effect on endothelial cells in VX2 tumors; the intra-arterial lipiodol (60°C) injections appeared to result in tumor capillary bed destruction.

In fact, although heat is toxic to tumor vascular endothelial cells, the precise effect of heat on the cells varies with the heat extent and duration. Severe or prolonged heat can alter intracellular proteins (such as collagen denaturation and lipid bilayer thawing) and induce cell death; if the heat is mild or short in duration, it can cause a series of adaptive genetic changes in the cells [22].

Heated lipiodol perfusion may not be sufficient to induce tumor vascular endothelial cell death but may cause a series of adaptive genetic or intracellular proteins changes such as the change of VEGFR expression in tumor vascular endothelial cells. Because the change of VEGFR expression is closely related to changes of endothelial cell morphology [23], we observed the morphological changes in tumor vascular endothelial cells by TEM after heated lipiodol infusion. We observed the decrease in VEGF and VEGFR mRNA and protein expression levels resulted from the tumor and endothelial cell responses to the heated lipiodol perfusion procedure, respectively. The decreased VEGF and VEGFR protein levels may be attributable to tumor angiogenesis inhibition.

### Preventing Tumor Growth After Heated Lipiodol Perfusion

Temperatures above 42.5°C are effective at suppressing tumor cell growth [24], but if the temperature is raised higher than 45°C, damage to normal liver cells becomes irreversible [24]. By understanding the non-homogeneous distribution of blood vessels, the cooling of tumor blood flow and tumor size differences, we conclude that the temperature of the heated lipiodol inside the tumors must be at least 43°C. In this study, firstly we gradually hand-injected under fluoroscopy to avoid the reflux during perfusion, secondly we used the percutaneous mathematical temperature recorder for monitoring the perfusion temperature, so the temperature of the heated lipiodol inside the liver tissues were not more than 45°C during the heated perfusion, and in our study the proximal normal vessels of rabbits still remain patent observed by DSA after heated perfusion.

Using immunohistochemistry, we observed low PCNA protein expression in tumor cells after heated lipiodol perfusion. According to our PCNA data, treatment with 60°C lipiodol markedly inhibited tumor growth in the rabbits with VX2 carcinomas compared to those treated with 37°C lipiodol.

It has been reported that tumor angiogenesis inhibition enhances anti-tumor efficacy [25]. Because anoxemic tumor cells have been shown to be more sensitive to heat than normal cells [26], [27] and the heated lipiodol can destroy the tumor capillary bed, the heated lipiodol can be detained in the tumor, and heat can be distributed to the neogenetic capillaries of the entire tumor. Theoretically, more heat can penetrate into the extracellular space of tumor cells to achieve an increased therapeutic effect. The longer tumor capillaries remained in the range of 43°C to 45°C, the more curative effect the heat had on tumor tissues, and then result in the inhibition of tumor angiogenesis and tumor cells’ growth.

### Limitations

Although we attempted to apply a percutaneous mathematical temperature recorder for monitoring, we were unable to accurately measure the temperature of the heated lipiodol in the entire tumor. The viscosity change of lipiodol with increased temperature and the impact of viscosity change on tumor such as biodistribution altering were not evaluated in our study, but will be addressed in future studies.

VX2 tumor is well known to develop necrosis which may alter the neovascularization rate, so in this study we selected VX2 tumors (14 days after implantation) for experimental treatment, because the VX2 tumors in this phase have a comparatively large tumor volume but small tumor necrosis and therefore have less effect on the neovascularization rate [10], [28].

## Conclusions

Effectively inhibiting tumor angiogenesis after TACE has been a research topic for some time. In this study, we found that decreased VEGF and VEGFR protein levels may be attributable to tumor angiogenesis inhibition in rabbits with VX2 cancer after arterial heated lipiodol infusion, but the clinical benefits of this approach need further evaluation, such as lipiodol viscosity change and biodistribution altering with increased temperature and so on, should be considered. Thus, this study could ultimately facilitate preclinical research, and we believe that heated lipiodol injection may effectively treat advanced or recurrent HCCs.

## References

[1] Vogl TJ, Trapp M, Schroeder H, Mack M, Schuster A, et al (2000) Transarterial chemoembolization for hepatocellular carcinoma: volumetric and morphologic CT criteria for assessment of prognosis and therapeutic success-results from a liver transplantation center. Radiology 214: 349–357. 10671580 10.1148/radiology.214.2.r00fe06349

[2] Llovet JM, Bruix J (2003) Systematic review of randomized trials for unresectable hepatocellular carcinoma: Chemoembolization improves survival. Hepatology 37: 429–442. 12540794 10.1053/jhep.2003.50047

[3] Achenbach T, Seifert JK, Pitton MB, Schunk K, Junginger T (2002) Chemoembolization for primary liver cancer. Eur J Surg Oncol 28: 37–41. 11869011 10.1053/ejso.2001.1181

[4] Marelli L, Stigliano R, Triantos C, Senzolo M, Cholongitas E, et al (2007) Transarterial therapy for hepatocellular carcinoma: which technique is more effective? A systematic review of cohort and randomized studies. Cardiovasc Intervent Radiol 30: 6–25. 17103105 10.1007/s00270-006-0062-3

[5] Lammer J, Malagari K, Vogl T, Pilleul F, Denys A, et al (2010) Prospective randomized study of doxorubicin-eluting-bead embolization in the treatment of hepatocellular carcinoma: results of the PRECISION V study. Cardiovasc Intervent Radiol 33: 41–52. 19908093 10.1007/s00270-009-9711-7 PMC2816794

[6] Poon RT, Ngan H, Lo CM, Liu CL, Fan ST, et al (2000) Transarterial chemoembolization for inoperable hepatocellular carcinoma and postresection intrahepatic recurrence. J Surg Oncol 73: 109–114. 10694648 10.1002/(sici)1096-9098(200002)73:2<109::aid-jso10>3.0.co;2-j

[7] Lo CM, Ngan H, Tso WK, Liu CL, Lam CM, et al (2002) Randomized controlled trial of transarterial lipiodol chemoembolization for unresectable hepatocellular carcinoma. Hepatology 35: 1164–1171. 11981766 10.1053/jhep.2002.33156

[8] Huang J, He X, Lin X, Zhang C, Li J (2000) Effect of preoperative transcatheter arterial chemoembolization on tumor cell activity in hepatocellular carcinoma. Chin Med J (Engl) 113: 446–448. 11776102

[9] Murray TG, Cicciarelli N, McCabe CM, Ksander B, Feuer W, et al (1997) In vitro efficacy of carboplatin and hyperthermia in a murine retinoblastoma cell line. Invest Ophthalmol Vis Sci 38: 2516–2522. 9375570

[10] Cao W, Wan Y, Liang ZH, Duan YY, Liu X, et al (2010) Heated lipiodol as an embolization agent for transhepatic arterial embolization in VX2 rabbit liver cancer model. Eur J Radiol 73: 412–419. 19091502 10.1016/j.ejrad.2008.11.001

[11] Cao W, Lu Q, Li JH, Zhou CX, Zhu J, et al (2011) Transcatheter arterial infusion with heated saline changes the vascular permeability of rabbit hepatic tumors. Acad Radiol 18: 1569–1576. 21968263 10.1016/j.acra.2011.08.010

[12] Cao W, Li JH, Feng DY, Wan Y, Liu YF, et al (2011) Effect of transarterial pulsed perfusion with heated saline on tumor vascular permeability in a rabbit VX2 liver tumor model. Eur J Radiol 81: e209–216. 21345630 10.1016/j.ejrad.2011.01.108

[13] Senger DR, Van de Water L, Brown LF, Nagy JA, Yeo KT, et al (1993) Vascular permeability factor (VPF, VEGF) in tumor biology. Cancer Metastasis Rev 12: 303–324. 8281615 10.1007/BF00665960

[14] Qin LX, Tang ZY (2002) The prognostic molecular markers in hepatocellular carcinoma. World J Gastroenterol 8: 385–392. 12046056 10.3748/wjg.v8.i3.385 PMC4656407

[15] Schmittgen TD, Zakrajsek BA, Mills AG, Gorn V, Singer MJ, et al (2000) Quantitative reverse transcription-polymerase chain reaction to study mRNA decay: comparison of endpoint and real-time methods. Anal Biochem 285: 194–204. 11017702 10.1006/abio.2000.4753

[16] Doyon D, Mouzon A, Jourde AM, Regensberg C, Frileux C (1974) [Hepatic, arterial embolization in patients with malignant liver tumours (author’s transl)]. Ann Radiol (Paris) 17: 593–603. 4142843

[17] Takayasu K, Shima Y, Muramatsu Y, Moriyama N, Yamada T, et al (1987) Hepatocellular carcinoma: treatment with intraarterial iodized oil with and without chemotherapeutic agents. Radiology 163: 345–351. 3031724 10.1148/radiology.163.2.3031724

[18] Hsieh MY, Lin ZY, Chuang WL (2011) Serial serum VEGF-A, angiopoietin-2, and endostatin measurements in cirrhotic patients with hepatocellular carcinoma treated by transcatheter arterial chemoembolization. Kaohsiung J Med Sci 27: 314–322. 21802642 10.1016/j.kjms.2011.03.008 PMC11916725

[19] Shim JH, Park JW, Kim JH, An M, Kong SY, et al (2008) Association between increment of serum VEGF level and prognosis after transcatheter arterial chemoembolization in hepatocellular carcinoma patients. Cancer Sci 99: 2037–2044. 19016764 10.1111/j.1349-7006.2008.00909.x PMC11158304

[20] Moyer HR, Delman KA (2008) The role of hyperthermia in optimizing tumor response to regional therapy. Int J Hyperthermia 24: 251–261. 18393003 10.1080/02656730701772480

[21] Ahrar K, Newman RA, Pang J, Vijjeswarapu MK, Wallace MJ, et al (2004) 2004 Dr. Gary J. Becker Young Investigator Award: Relative thermosensitivity of cytotoxic drugs used in transcatheter arterial chemoembolization. J Vasc Interv Radiol 15: 901–905. 15361556 10.1097/01.RVI.0000136829.36643.ED

[22] Dikomey E, Franzke J (1992) Effect of heat on induction and repair of DNA strand breaks in X-irradiated CHO cells. Int J Radiat Biol 61: 221–233. 1351910 10.1080/09553009214550851

[23] Schuh AC, Faloon P, Hu QL, Bhimani M, Choi K (1999) In vitro hematopoietic and endothelial potential of flk-1(−/−) embryonic stem cells and embryos. Proc Natl Acad Sci U S A 96: 2159–2164. 10051611 10.1073/pnas.96.5.2159 PMC26753

[24] Feldman ED, Wu PC, Beresneva T, Helsabeck C, Rodriguez M, et al (2004) Treatment of patients with unresectable primary hepatic malignancies using hyperthermic isolated hepatic perfusion. J Gastrointest Surg 8: 200–207. 15036196 10.1016/j.gassur.2003.11.005

[25] Ferrara N (2005) VEGF as a therapeutic target in cancer. Oncology 69 Suppl 311–16. 16301831 10.1159/000088479

[26] Nesher E, Greenberg R, Avital S, Skornick Y, Schneebaum S (2007) Cytoreductive surgery and intraperitoneal hyperthermic chemotherapy in peritoneal carcinomatosis. Isr Med Assoc J 9: 787–790. 18085034

[27] Muraoka A, Takeda S, Matsui M, Shimizu T, Tohnai I, et al (2004) Experimental study of a novel thermotherapy for hepatocellular carcinoma using a magnesium ferrite complex powder that produces heat under a magnetic field. Hepatogastroenterology 51: 1662–1666. 15532799

[28] Ramirez LH, Zhao Z, Rougier P, Bognel C, Dzodic R, et al (1996) Pharmacokinetics and antitumor effects of mitoxantrone after intratumoral or intraarterial hepatic administration in rabbits. Cancer Chemother Pharmacol 37: 371–376. 8548884 10.1007/s002800050399

